# Image-Domain GAN Denoising for Sn100 kVp Ultra-Low-Dose Chest CT: A Retrospective Paired Image-Quality Study

**DOI:** 10.3390/diagnostics16182988

**Published:** 2026-09-15

**Authors:** Kaiqing Yao, Liang Lv, Xue Jiang, Yang Li, Guangpeng Zhang, Wangjia Li, Yineng Zheng, Zhiyuan Zhang, Zhiwei Zhang, Xinyou Li, Fajin Lv

**Affiliations:** 1 Department of Radiology, The First Affiliated Hospital of Chongqing Medical University, Chongqing 400016, China; kaiqingyao@163.com (K.Y.); 204510@hospital.cqmu.edu.cn (X.J.); 18883938636@163.com (W.L.); yinengzheng@cqmu.edu.cn (Y.Z.); zhangzhiyuan422@126.com (Z.Z.); zhangzhiweicqmu@163.com (Z.Z.); lixinyou666@163.com (X.L.); 2 Department of Radiology, Chongqing Academy of Medical Sciences, Chongqing General Hospital, Chongqing University, Chongqing 400044, China; lianglv@cqu.edu.cn; 3 Department of Physical Algorithm, Sinovision Technologies (Beijing) Co., Ltd., Beijing 100176, China; yang.li@sinovision-tech.com; 4 Department of Safety Assessment, Chongqing Changming Safety Technology Consulting Co., Ltd., Chongqing 401122, China; zhangguangpeng@gmail.com

**Keywords:** ultra-low-dose CT, image-domain denoising, generative adversarial network, image quality, radiation dose

## Abstract

**Background:** Ultra-low-dose (ULD) chest CT can reduce radiation exposure but may compromise image quality. We evaluated image-domain generative adversarial network (GAN)-based denoising at low-dose (LD) and ULD levels, focusing on ULD-AiR versus LD-ADMIRE S3. **Methods:** In this single-center retrospective paired study, 262 participants underwent LD and ULD chest CT on the same scanner. Images reconstructed with Advanced Modeled Iterative Reconstruction at strength 3 (ADMIRE S3) were post-processed with AiR Denoising v1.0, yielding four series. Objective metrics, including signal-to-noise ratio (SNR) and contrast-to-noise ratio (CNR), and 5-point subjective ratings were compared within dose levels and between ULD-AiR and LD-ADMIRE S3. Exploratory regression assessed associations of ULD-to-LD SNR log-ratios with the volume CT dose index (CTDI_vol_) log-ratio and anthropometric variables. **Results:** The median paired reduction in estimated effective dose from LD to ULD CT was 46.5%. Within each dose level, AiR reduced image noise and increased SNR, CNR, and subjective scores. Compared with LD-ADMIRE S3, ULD-AiR showed lower image noise and higher SNR/CNR in the lung, aorta, and muscle; liver findings were less consistent, whereas vertebral metrics were less favorable. Lung-parenchyma scores did not differ significantly, mediastinal soft-tissue scores favored ULD-AiR, and overall image-noise scores favored LD-ADMIRE S3. Exploratory regression identified CTDI_vol_ log-ratio associations with aortic and muscle SNR log-ratios; some anthropometric associations were sensitive to an influential observation. **Conclusions:** Image-domain GAN denoising improved several objective and subjective image-quality metrics at both dose levels. With a median paired reduction of 46.5% in estimated effective dose, ULD-AiR showed tissue- and endpoint-specific image-quality differences relative to LD-ADMIRE S3. These findings do not establish diagnostic or screening equivalence.

## 1. Introduction

Low-dose (LD) chest CT has been shown to reduce lung cancer mortality in high-risk populations [1,2]. However, cumulative radiation exposure remains a concern with repeated screening examinations [3,4]. Ultra-low-dose (ULD) CT, often implemented with spectral shaping techniques such as tin filtration, aims to achieve submillisievert examinations while preserving clinically acceptable image quality [5,6]. At these very low doses, quantum noise and loss of low-contrast detail can compromise image quality with filtered back projection (FBP) and conventional iterative reconstruction (IR) [7,8]. Deep-learning image reconstruction (DLIR) has been reported to reduce image noise and improve signal-to-noise ratio (SNR) and contrast-to-noise ratio (CNR) compared with conventional IR [9,10]. Recent studies have shown that DLIR can improve image quality and, in some studies, lesion detection in submillisievert or ULD chest CT compared with conventional IR [11,12,13]. More recently, image-domain deep-learning post-processing has been evaluated for ULD CT, with improvements in image quality and pulmonary nodule detection reported using LD CT as a reference [14]. Nevertheless, the available evidence remains heterogeneous across scanner platforms, algorithmic domains (projection versus image domain), reconstruction strengths, endpoints (image quality versus detectability), and study designs [15]. In particular, the effects of image-domain denoising on objective and subjective image-quality metrics at ULD dose levels, as well as differences relative to LD reference images, remain incompletely characterized.

Many studies have compared DLIR with IR at a single dose level or in heterogeneous cohorts, limiting characterization of image-quality changes across dose levels [9,10,11,12,15]. In addition, tissue-specific factors associated with image-quality changes across dose levels remain incompletely understood [15]. Image-domain deep-learning post-processing offers a distinct approach because it can be applied to reconstructed image data without requiring access to raw projection data, and this approach has been evaluated in thin-section low-dose or ULD chest CT [16,17]. However, paired evidence directly characterizing image-quality changes between LD and ULD examinations remains relatively limited [15].

To address these gaps, we conducted a retrospective paired image-quality study in a routine health check-up setting. Participants had undergone separate LD and ULD chest CT examinations for lung cancer screening on the same scanner. We aimed to characterize differences in objective and subjective image-quality metrics between ULD-AiR and LD images reconstructed with Advanced Modeled Iterative Reconstruction at strength 3 (ADMIRE S3). We also evaluated within-dose differences between AiR-processed and corresponding ADMIRE S3 images at both LD and ULD dose levels and explored associations of the ULD-to-LD SNR log-ratios with the volume CT dose index (CTDI_vol_) log-ratio, anthropometric factors, examination interval, and examination order.

## 2. Materials and Methods

### 2.1. Study Population

This retrospective study was approved by the Ethics Committee of the First Affiliated Hospital of Chongqing Medical University (approval No. 2025-606-01; approved on 2 September 2025), with a waiver of informed consent. Consecutive adults who underwent two chest CT examinations for lung cancer screening as an optional component of routine health check-ups on the same scanner (SOMATOM Force; Siemens Healthineers, Forchheim, Germany) between December 2020 and February 2025 were identified. Inclusion criteria were as follows: (i) availability of the original reconstructed thin-section Digital Imaging and Communications in Medicine (DICOM) series for both examinations; and (ii) one examination with an estimated effective dose in the predefined LD range (0.5–1.0 mSv) and the other in the ULD range (<0.5 mSv). Exclusion criteria were as follows: (i) severe artifacts, including marked respiratory motion on either examination; (ii) image quality insufficient for reliable region of interest (ROI) measurements.

**Figure 1:**
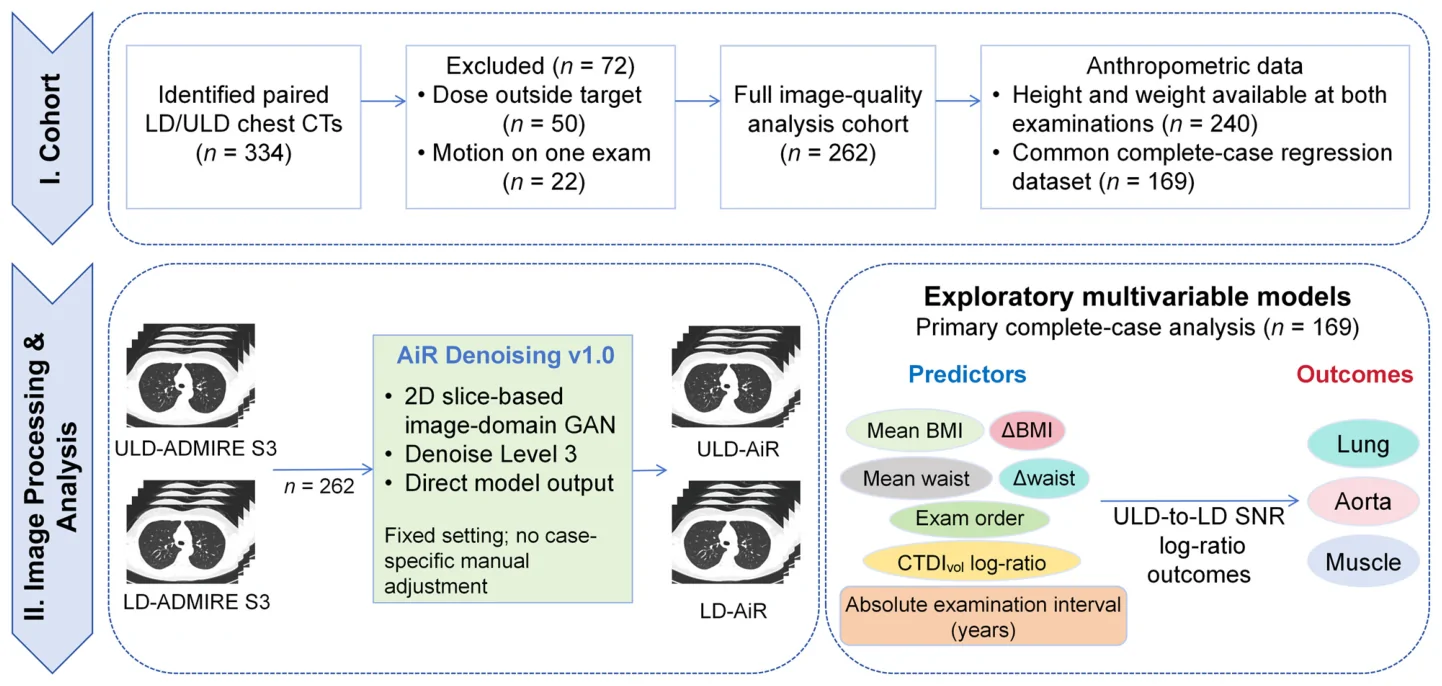
Study flow, image-processing workflow, and exploratory multivariable analysis. Δ denotes ULD minus LD. ADMIRE—Advanced Modeled Iterative Reconstruction; AiR—AiR Denoising; BMI—body mass index; CTDI_vol_—volume CT dose index; GAN—generative adversarial network; LD—low dose; SNR—signal-to-noise ratio; ULD—ultra-low dose.

A total of 334 adults were screened. Fifty were excluded because the paired examinations did not meet the predefined LD/ULD dose criteria: 46 had both examinations below 0.5 mSv, one had both examinations between 0.5 and 1.0 mSv, and three had one examination above 1.0 mSv. An additional 22 participants were excluded because of marked respiratory motion on one examination, leaving 262 adults with paired LD and ULD examinations for analysis. Participant characteristics, including sex, age, height, weight, body mass index (BMI), waist circumference, hip circumference, and waist-to-hip ratio, were retrieved from the electronic medical record. For descriptive characterization of the cohort, pulmonary nodule status and the largest documented pulmonary nodule diameter were retrospectively extracted from the formal radiology report of the earlier examination in each pair, defined as the index examination. An overview of the study design and analysis framework is provided in Figure 1.

### 2.2. CT Acquisition, Image Reconstruction, and AiR Post-Processing

All CT examinations were performed on a third-generation dual-source CT scanner (SOMATOM Force; Siemens Healthineers, Forchheim, Germany) using tin-filtered spectral shaping with single-source spiral acquisition. Scans were acquired in the caudocranial direction during a full-inspiration breath-hold from the lung apices through the costophrenic angles. For LD CT, the tube potential was Sn100 kVp with CARE Dose 4D automatic exposure control, a quality reference mAs of 400, and a gantry rotation time of 0.50 s. For ULD CT, the tube potential was also Sn100 kVp with CARE Dose 4D, a quality reference mAs of 200, and a gantry rotation time of 0.28 s. For both protocols, detector collimation was 192 × 0.6 mm, pitch was 1.5, and the image matrix was 512 × 512. Images from both protocols were reconstructed using Advanced Modeled Iterative Reconstruction at strength 3 (ADMIRE S3) with a Br40 kernel, a 1.0 mm slice thickness, and a 0.7 mm increment.

De-identified thin-section axial DICOM series were processed offline using AiR Denoising v1.0 (Sinovision Technologies, Beijing, China). AiR is a two-dimensional, slice-based image-domain denoising model with a generative adversarial network (GAN) architecture, comprising a modified residual encoder–decoder convolutional neural network (RED-CNN) generator and a convolutional neural network (CNN) discriminator derived from the Wasserstein generative adversarial network (WGAN) framework. All study images were processed using the fixed Denoise Level 3 setting, corresponding to the direct model output, without case-specific manual adjustment.

### 2.3. Radiation Dose Assessment

Radiation output was characterized using CTDI_vol_ and dose-length product (DLP), which were extracted from the scanner-generated dose reports. The reported CTDI_vol_ values were referenced to the 32 cm body CTDI phantom. Estimated effective dose was calculated as DLP × 0.014 mSv·mGy^−1^·cm^−1^ using a chest-specific conversion coefficient [18].

### 2.4. Image Quality Assessment

Image quality was assessed on a dedicated workstation using uWS-MR medical image processing software, version R005.31.1.1535882 (United Imaging Healthcare, Shanghai, China), by three readers. Reader A (dual-qualified radiographer–radiologist; 13 years of experience) performed both objective measurements and subjective ratings; Reader B (radiographer; 16 years of experience) performed objective measurements; and Reader C (radiologist; 7 years of experience) performed subjective ratings. Before formal evaluation, Readers A and B underwent joint calibration to standardize ROI placement and measurement procedures, whereas Readers A and C underwent joint calibration to standardize application of the predefined subjective scoring criteria, using a subset of study cases.

#### 2.4.1. Objective Image Quality Assessment

Readers A and B independently performed quantitative measurements on four image series (LD-ADMIRE S3, ULD-ADMIRE S3, LD-AiR, and ULD-AiR). The readers were aware of the dose level and image-processing method of each series but had no access to each other’s measurements. LD and ULD examinations were measured separately. Within each examination, the ADMIRE S3 and corresponding AiR series were evaluated as a paired image set, and ROI position and size were directly transferred between the paired series to ensure spatial consistency. Standardized circular ROIs (approximately 100 mm^2^) were placed to record the mean attenuation value in Hounsfield units (HU) and corresponding standard deviation (SD), with SD used as a measure of image noise. ROIs were placed in normal lung parenchyma adjacent to the right major fissure, at the center of the descending aortic lumen (avoiding the wall), in the right subscapularis muscle, in segment VII of the right hepatic lobe, and at the center of the T12 vertebral body. A background-air ROI was placed in extracorporeal air 3–5 cm anterior to the sternum [10,19]. For CNR calculation, each evaluated tissue ROI served as the target ROI, whereas the extracorporeal air ROI served as the background. Within each examination, ROI position and size were directly transferred from the ADMIRE S3 series to the corresponding AiR series. Between the separate LD and ULD examinations, ROIs were placed at the same predefined anatomical locations using approximately the same ROI size.

To account for negative attenuation values in the lung, SNR was expressed as an absolute magnitude. SNR and CNR were calculated using formulations previously applied in CT image-quality studies [10,19,20], as follows:

(1) $$\mathrm{SNR}=\frac{\left|{HU}_{\mathrm{ROI}}\right|}{{\mathrm{SD}}_{\mathrm{ROI}}}$$

(2) $$\mathrm{CNR}=\frac{2(HU_{\mathrm{Target}}-HU_{\mathrm{Background}\;\mathrm{air}}{)}^{2}}{SD_{\mathrm{Target}}^{2}+SD_{\mathrm{Background}\;\mathrm{air}}^{2}}$$

where HU_ROI_ and SD_ROI_ are the mean attenuation value and corresponding standard deviation within the ROI, respectively; HU_Target_ and HU_Background air_ are the mean attenuation values of the target tissue and background air, respectively; and SD_Target_ and SD_Background air_ are their corresponding standard deviations.

#### 2.4.2. Subjective Image Quality Assessment

Readers A and C independently evaluated all four image series (LD-ADMIRE S3, ULD-ADMIRE S3, LD-AiR, and ULD-AiR), which were presented in randomized order. Series labels identifying the dose level and image-processing method were concealed, and the readers had no access to clinical information or each other’s scores. Images were initially displayed using standard lung-window settings (window width, 1200 HU; window level, −600 HU) and mediastinal-window settings (window width, 350 HU; window level, 40 HU), with free adjustment of window width and level permitted during evaluation. Lung parenchyma, mediastinal soft tissue, and overall image noise were assessed on 5-point Likert scales (1 = unacceptable, 2 = suboptimal, 3 = acceptable, 4 = good, 5 = optimal) [19,21]. Prespecified score anchors (operational definitions) are provided in Supplementary Table S1.

### 2.5. Statistical Analysis

Statistical analyses were performed using IBM SPSS Statistics, version 32.0 (IBM Corp., Armonk, NY, USA) and R, version 4.6.0 (R Foundation for Statistical Computing, Vienna, Austria). Continuous variables were summarized as mean ± standard deviation or median [interquartile range (IQR)], as appropriate. Paired comparisons were performed using paired *t*-tests or Wilcoxon signed-rank tests, as appropriate. Three prespecified paired comparisons were evaluated: ULD-AiR versus ULD-ADMIRE S3, ULD-AiR versus LD-ADMIRE S3, and LD-AiR versus LD-ADMIRE S3. Benjamini–Hochberg false discovery rate (FDR) correction was applied separately to the objective and subjective tests within each comparison, yielding six multiplicity families in total. Each objective family comprised 44 reader- and tissue-specific tests across CT attenuation, image noise, SNR, and CNR, whereas each subjective family comprised six reader- and domain-specific tests. Subjective scores were summarized as median [interquartile range] and compared using two-sided Wilcoxon signed-rank tests. Paired effect estimates were reported as mean differences with *t*-based 95% confidence intervals (CIs) for paired *t* tests and as median differences with 10,000-resample paired-case percentile bootstrap 95% CIs for Wilcoxon comparisons, including ordinal ratings. For these objective and subjective image-quality comparisons, FDR-adjusted *p*-values < 0.05 were considered statistically significant.

Inter-reader reliability for objective measurements was assessed using single-measure intraclass correlation coefficients (ICCs) from a two-way mixed-effects consistency model with 95% CIs [22]. Subjective agreement was assessed using linear-weighted Cohen’s κ with 95% percentile CIs obtained from 10,000 paired-case bootstrap resamples.

Exploratory tissue-specific multivariable linear regression models were fitted for the lung, aorta, and muscle. Two ULD-to-LD SNR percent log-ratio outcomes were analyzed [23]:

$$100\times ln(\frac{{SNR}_{ULD-ADMIRE\;S3}}{{SNR}_{LD-ADMIRE\;S3}})$$

and

$$100\times ln(\frac{{SNR}_{ULD-AiR}}{{SNR}_{LD-ADMIRE\;S3}}).$$

The primary analysis included 169 complete cases. Covariates were mean BMI, ΔBMI, mean waist circumference, Δwaist circumference, absolute examination interval, examination order, and the CTDI_vol_ log-ratio; changes were defined as ULD minus LD; examination order was coded as ULD first = 1, and LD first = 0; and the CTDI_vol_ log-ratio was defined as $ln(\frac{{CTDI}_{vol}(ULD)}{{CTDI}_{vol}(LD)})$. Multicollinearity, model specification, and influential observations were assessed using variance inflation factors, residual diagnostics, the Ramsey regression equation specification error test (RESET), and influence diagnostics.

For sensitivity analyses, the same models were refitted using 172 complete cases and, separately, after multiple imputation of missing anthropometric data in all 262 participants (50 imputations using predictive mean matching), with estimates pooled using Rubin’s rules. Targeted case-deletion analyses were used for influence assessment only. All regression *p*-values were two-sided, unadjusted, and exploratory.

## 3. Results

### 3.1. Basic Characteristics and Radiation Dose

The analysis included 262 participants, each contributing one eligible pair of LD and ULD chest CT examinations. At the time of the LD examination, the median age was 49 years (range, 22–86 years), and the cohort included 146 men and 116 women. Anthropometric data were not available at both examinations for all participants; the common complete-case analysis set used for the primary exploratory regression analyses comprised 169 participants.

Based on the formal radiology report of the index examination, pulmonary nodules were documented in 241 of 262 participants (92.0%), whereas no pulmonary nodule was documented in 21 (8.0%). Among the 241 participants with report-documented pulmonary nodules, the largest nodule diameter was reported in 230 (95.4%); the median largest diameter was 4 mm [IQR, 3–5 mm] (range, 2–16 mm).

ULD CT had substantially lower radiation output than LD CT. Median CTDI_vol_ was 0.68 [0.59, 0.79] mGy for ULD CT and 1.28 [1.15, 1.43] mGy for LD CT, corresponding to a median paired reduction of 48.3% [40.8%, 53.0%]. Median estimated effective dose was 0.354 [0.304, 0.410] mSv and 0.629 [0.570, 0.701] mSv, respectively, corresponding to a median paired reduction of 46.5% [38.7%, 51.5%] (both *p* < 0.001; Table 1).

### 3.2. Objective Image-Quality Analysis

CT attenuation was generally stable across dose levels and image-processing methods. Within each dose level, statistically significant differences between AiR and ADMIRE S3 were small in magnitude, with absolute paired differences of less than 1 HU. In the cross-dose comparison between ULD-AiR and LD-ADMIRE S3, no statistically significant differences were observed in the lung, aorta, muscle, liver, or vertebral body for either reader after FDR correction. A small difference in background-air attenuation remained significant for Reader B after FDR correction. Paired CT-attenuation effect estimates with 95% CIs are provided in Supplementary Table S2, whereas the corresponding image-series distributions, FDR-adjusted *p*-values, and inter-reader reliability results are provided in Supplementary Table S3.

**Figure 2:**
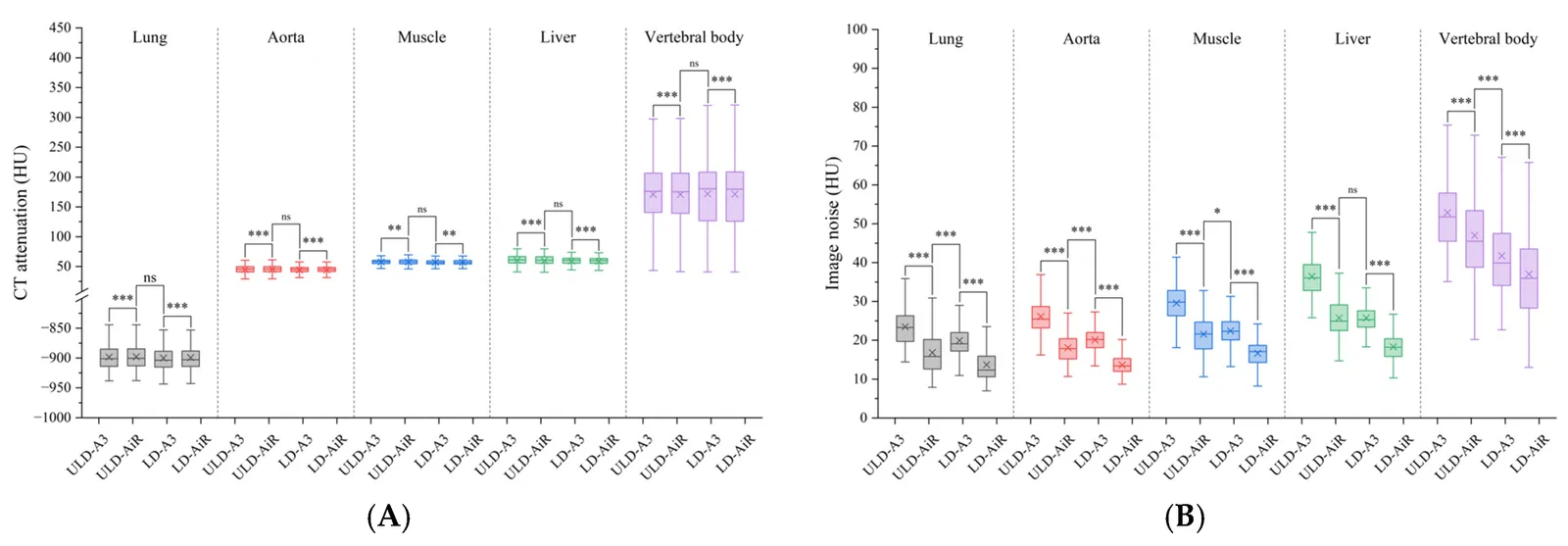

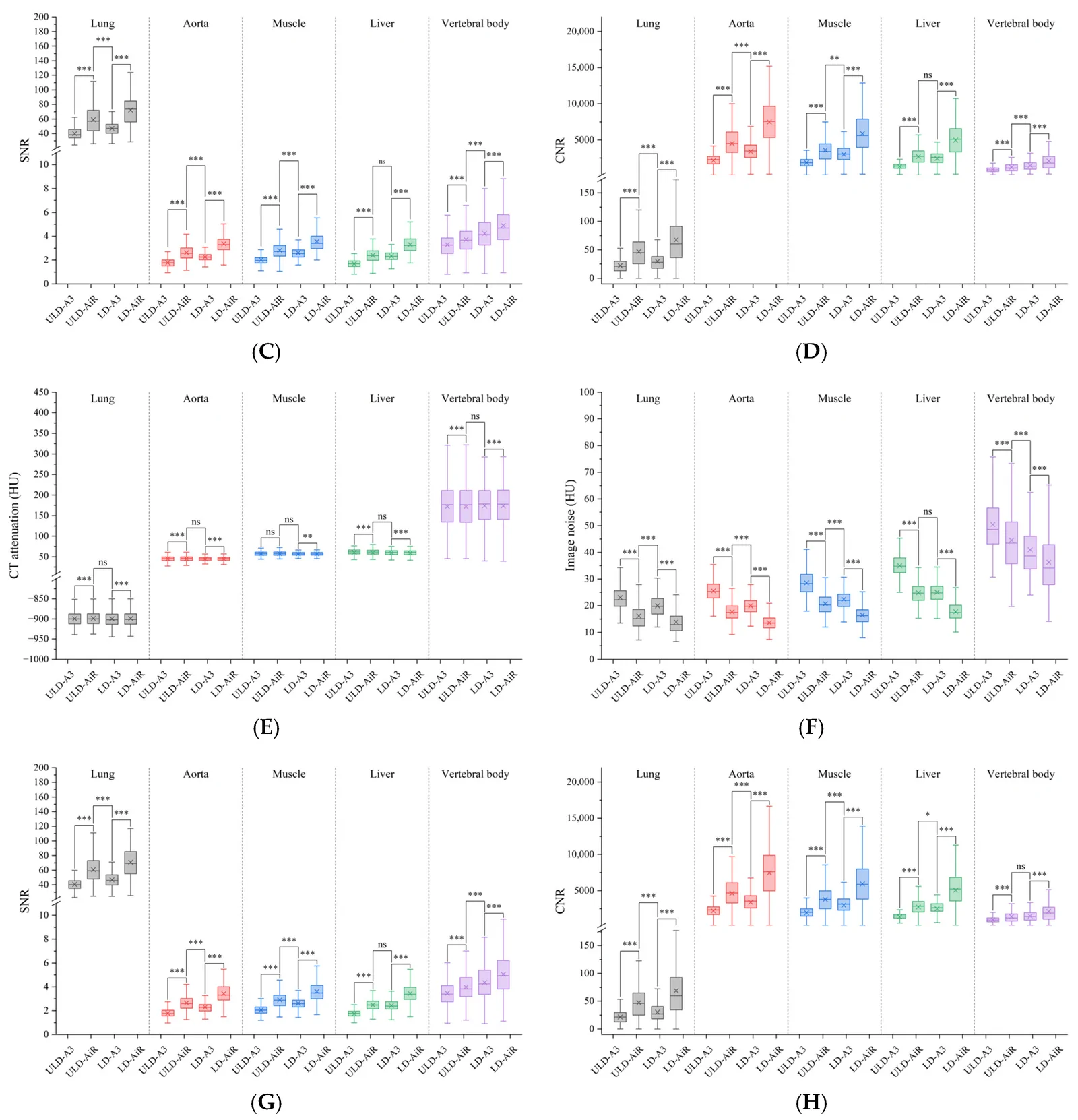
Distributions of objective image-quality metrics across the four-image series. (**A**–**D**) CT attenuation, image noise, signal-to-noise ratio (SNR), and contrast-to-noise ratio (CNR), respectively, measured by Reader A; (**E**–**H**) the corresponding measurements by Reader B. Colors indicate the anatomical regions: lung (gray), aorta (red), muscle (blue), liver (green), and vertebral body (purple). In the axis labels, A3 denotes ADMIRE at strength level 3 (ADMIRE S3). Lung SNR values are presented as absolute magnitudes. Statistical annotations indicate the three prespecified paired comparisons: ULD-AiR versus ULD-ADMIRE S3, ULD-AiR versus LD-ADMIRE S3, and LD-AiR versus LD-ADMIRE S3. “ns” indicates an FDR-adjusted *p*-value ≥ 0.05; “*”, “**”, and “***” indicate FDR-adjusted *p*-values < 0.05, <0.01, and <0.001, respectively. The × symbol indicates the mean. ADMIRE—Advanced Modeled Iterative Reconstruction; AiR—AiR Denoising; CT—computed tomography; FDR—false discovery rate; LD—low dose; ULD—ultra-low dose.

Within each dose level, AiR consistently reduced image noise and increased SNR and CNR across the evaluated tissues for both readers, with all corresponding comparisons remaining significant after FDR correction. In the cross-dose comparison between ULD-AiR and LD-ADMIRE S3, image noise was lower in the lung, aorta, muscle, and background air, similar in the liver, and higher in the vertebral body. For lung image noise, the paired differences were −3.6 HU (95% CI, −4.35 to −2.90 HU) for Reader A and −4.4 HU (95% CI, −4.90 to −3.85 HU) for Reader B. SNR was higher in the lung, aorta, and muscle, similar in the liver, and lower in the vertebral body. CNR was higher in the lung, aorta, and muscle for both readers. Liver CNR was higher for Reader B but not for Reader A after FDR correction, whereas vertebral-body CNR was lower for Reader A and did not differ significantly for Reader B. Overall quantitative distributions are shown in Figure 2, with detailed image-noise, SNR, and CNR results, including paired effect estimates with 95% CIs, provided in Supplementary Tables S4–S6.

**Figure 3:**
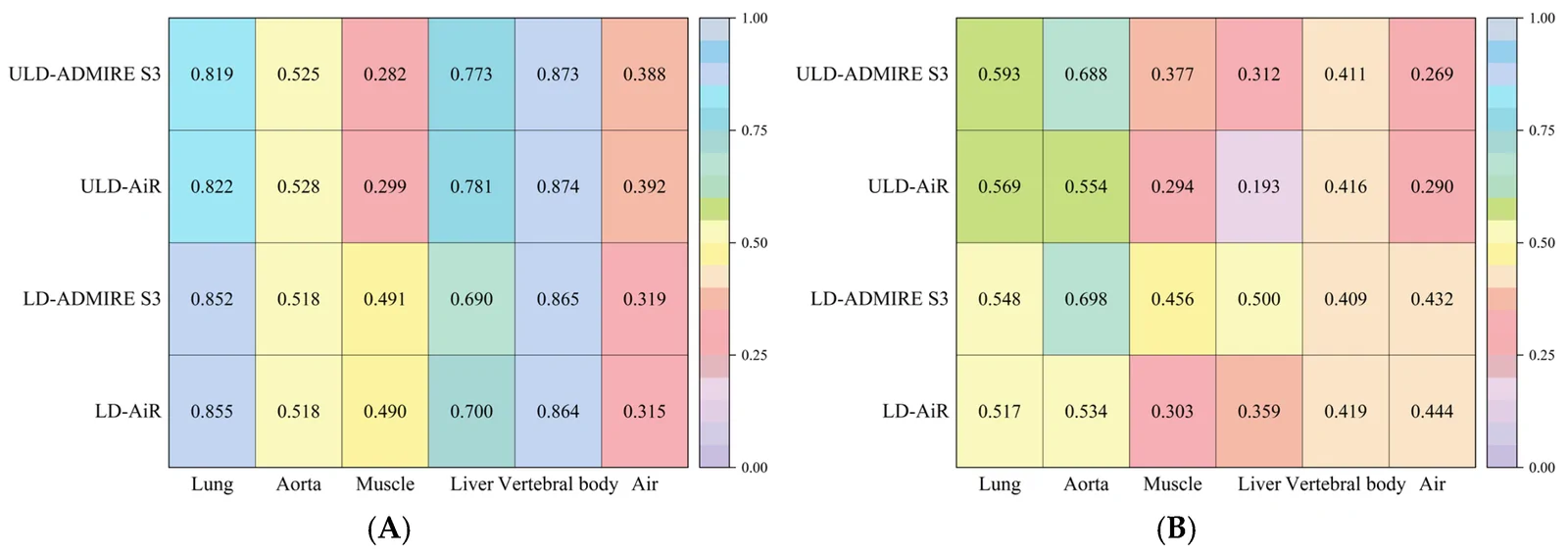
Inter-reader reliability of objective image-quality measurements. Heatmaps show single-measure intraclass correlation coefficient (ICC) estimates for (**A**) CT attenuation and (**B**) image noise across the four-image series and six evaluated regions. ICCs were derived using a two-way mixed-effects consistency model. Cells display ICC point estimates; corresponding 95% confidence intervals are provided in Supplementary Tables S3 and S4. In the axis labels, Air denotes background air. ADMIRE—Advanced Modeled Iterative Reconstruction; AiR—AiR Denoising; CT—computed tomography; ICC—intraclass correlation coefficient; LD—low dose; ULD—ultra-low dose.

Inter-reader reliability varied by tissue and quantitative metric and was generally higher for CT attenuation than for image-noise measurements. Attenuation measurements showed relatively higher reliability in the lung, liver, and vertebral body, whereas lower reliability was observed for several muscle and background-air measurements. Image-noise measurements showed greater inter-reader variability, particularly in the liver and muscle. ICC estimates with 95% CIs are summarized in Figure 3 and Supplementary Tables S3 and S4.

### 3.3. Subjective Image-Quality Analysis

Within each dose level, AiR-processed images received higher scores than the corresponding ADMIRE S3 images for lung parenchyma, mediastinal soft tissue, and overall image noise, with all comparisons remaining significant after FDR correction. In the cross-dose comparison between ULD-AiR and LD-ADMIRE S3, lung parenchyma scores showed no significant difference for either reader after FDR correction, whereas mediastinal soft-tissue scores favored ULD-AiR and overall image-noise scores favored LD-ADMIRE S3. Effect directions were concordant between the two readers. Detailed subjective scores, paired effect estimates with 95% CIs, and inter-reader agreement are provided in Supplementary Table S7.

**Figure 4:**
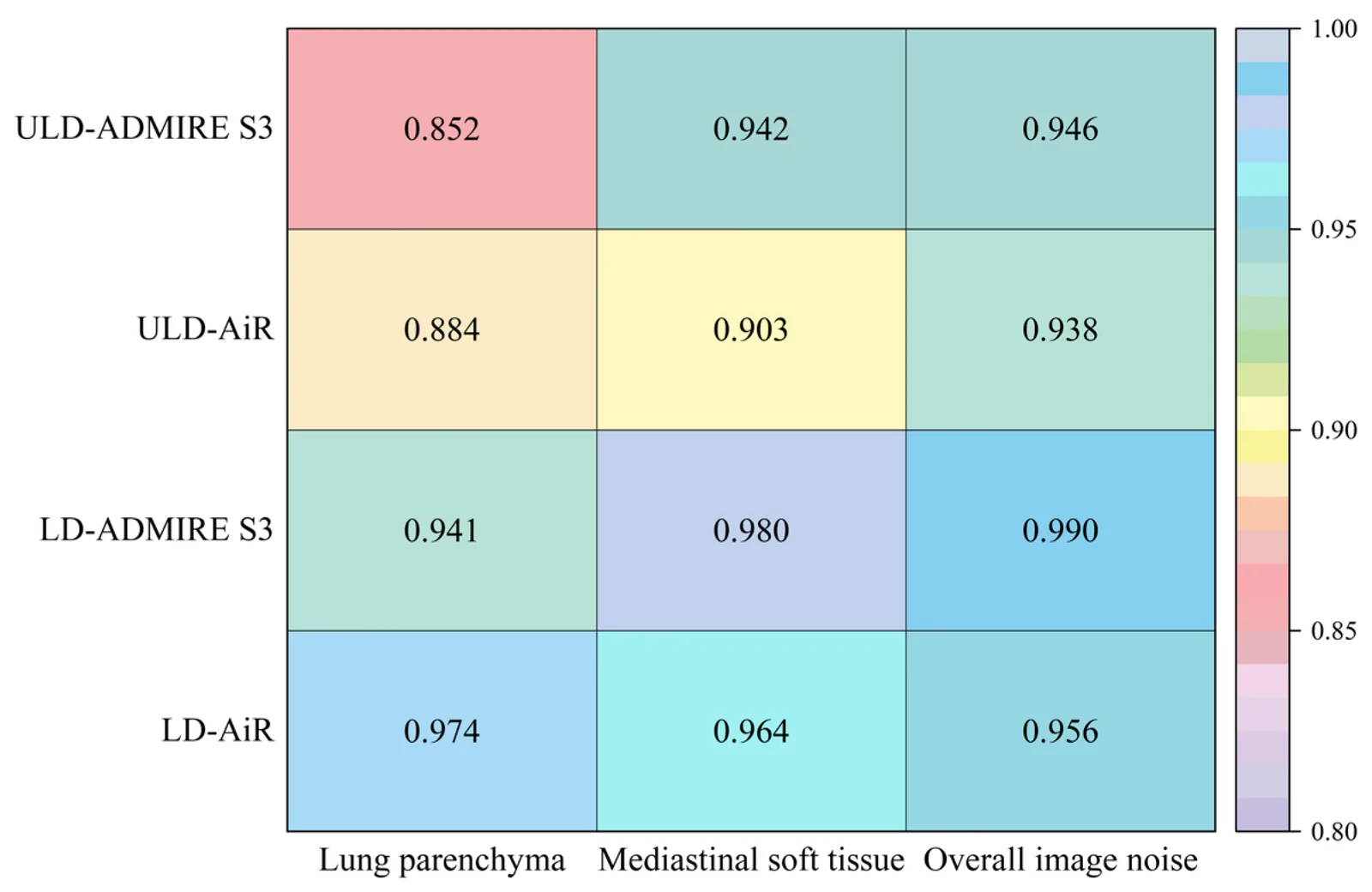
Inter-reader agreement for subjective image-quality ratings. The heatmap shows linear-weighted Cohen’s κ estimates between Readers A and C across the four-image series and three subjective image-quality domains: lung parenchyma, mediastinal soft tissue, and overall image noise. Cells display weighted κ point estimates; corresponding percentile bootstrap 95% confidence intervals based on 10,000 paired-case resamples are provided in Supplementary Table S7. ADMIRE—Advanced Modeled Iterative Reconstruction; AiR—AiR Denoising; LD—low dose; ULD—ultra-low dose.

**Figure 5:**
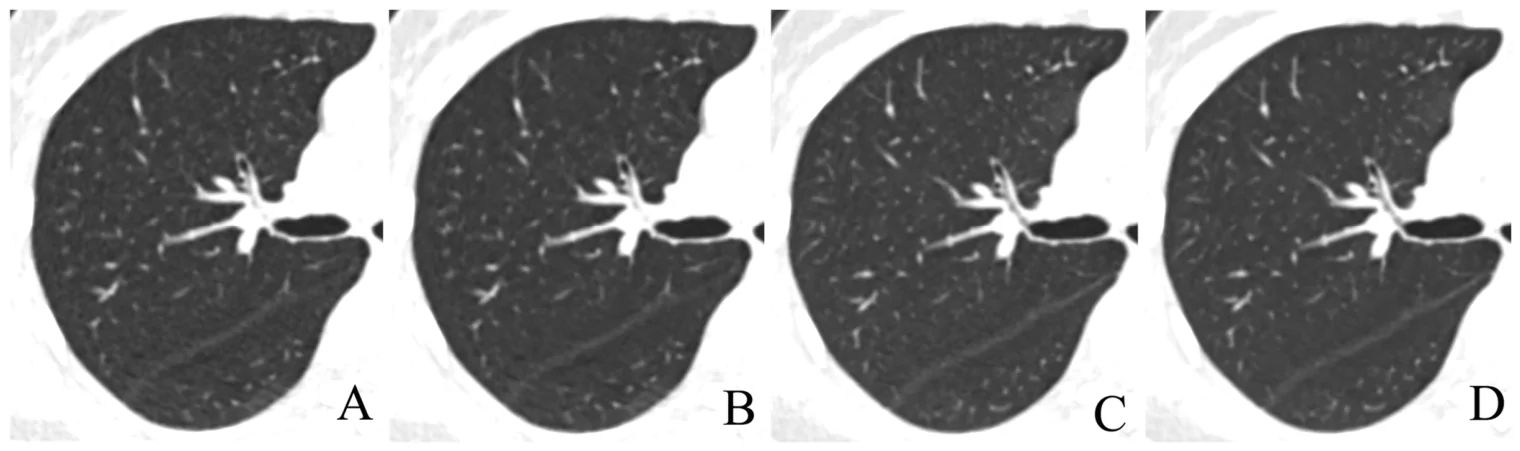
Representative axial lung-window CT images from one participant at the ULD and LD dose levels. Panels show (**A**) ULD-ADMIRE S3, (**B**) ULD-AiR, (**C**) LD-ADMIRE S3, and (**D**) LD-AiR images. All images are displayed using the same lung-window setting. The images were cropped and enlarged for display to facilitate visual comparison of image quality. ADMIRE—Advanced Modeled Iterative Reconstruction; AiR—AiR Denoising; CT—computed tomography; LD—low dose; ULD—ultra-low dose.

**Figure 6:**
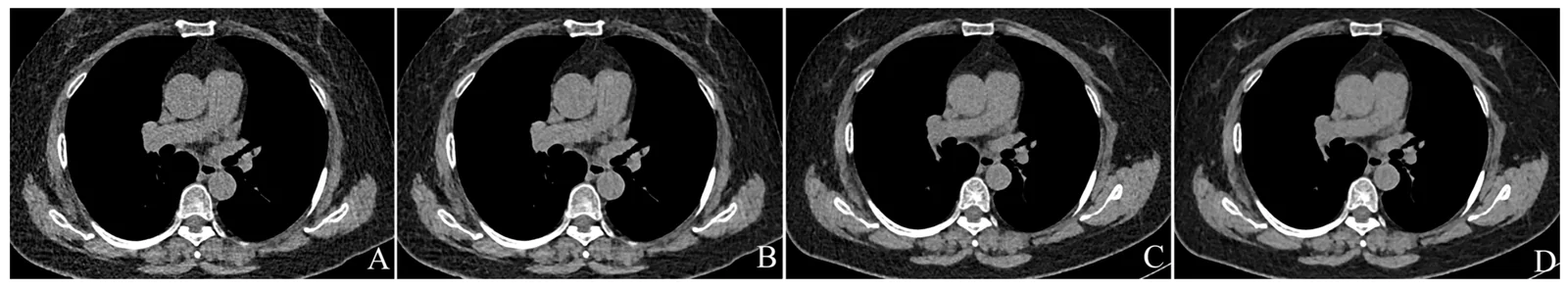
Representative axial mediastinal-window CT images from one participant at the ULD and LD dose levels. Panels show (**A**) ULD-ADMIRE S3, (**B**) ULD-AiR, (**C**) LD-ADMIRE S3, and (**D**) LD-AiR images. The images were cropped and enlarged for display to facilitate visual comparison of image quality. All images are displayed using the same mediastinal window setting. ADMIRE—Advanced Modeled Iterative Reconstruction; AiR—AiR Denoising; CT—computed tomography; LD—low dose; ULD—ultra-low dose.

Inter-reader agreement was high across image series and rating domains. Linear-weighted Cohen’s κ ranged from 0.852 to 0.974 for lung parenchyma, from 0.903 to 0.980 for mediastinal soft tissue, and from 0.938 to 0.990 for overall image noise. Weighted κ estimates with bootstrap 95% CIs are summarized in Figure 4 and Supplementary Table S7. Representative CT images are shown in Figure 5 and Figure 6.

### 3.4. Multivariable Linear Regression

Exploratory multivariable regression analyses were performed for two SNR log-ratio outcomes: ULD-ADMIRE S3 relative to LD-ADMIRE S3 and ULD-AiR relative to LD-ADMIRE S3. An exploratory, unadjusted comparison of participants included in the primary complete-case regression cohort (*n* = 169) and those not included under the original complete-case rule (*n* = 93) showed no statistically clear differences in age, sex, or LD CTDI_vol_. Differences were observed in examination interval, examination order, ULD CTDI_vol_, the CTDI_vol_ log-ratio, ULD estimated effective dose, and ULD-ADMIRE S3 lung SNR, indicating an observed selection pattern. In the primary complete-case analysis (*n* = 169), the CTDI_vol_ log-ratio was positively associated with the aortic SNR log-ratio for both ULD-ADMIRE S3 (*B* = 28.827, 95% CI, 2.957 to 54.696; *p* = 0.029) and ULD-AiR (*B* = 34.329, 95% CI, 4.685 to 63.974; *p* = 0.024), and with the muscle SNR log-ratio for ULD-ADMIRE S3 (*B* = 41.468, 95% CI, 17.434 to 65.502; *p* < 0.001) and ULD-AiR (*B* = 54.109, 95% CI, 26.154 to 82.065; *p* < 0.001). For ULD-ADMIRE S3, ΔBMI was inversely associated with the lung SNR log-ratio (*B* = −5.069, 95% CI, −9.736 to −0.402; *p* = 0.033). Although ΔBMI was also inversely associated with the muscle SNR log-ratio in both comparisons, these associations were sensitive to an influential observation and were therefore not considered robust.

**Figure 7:**
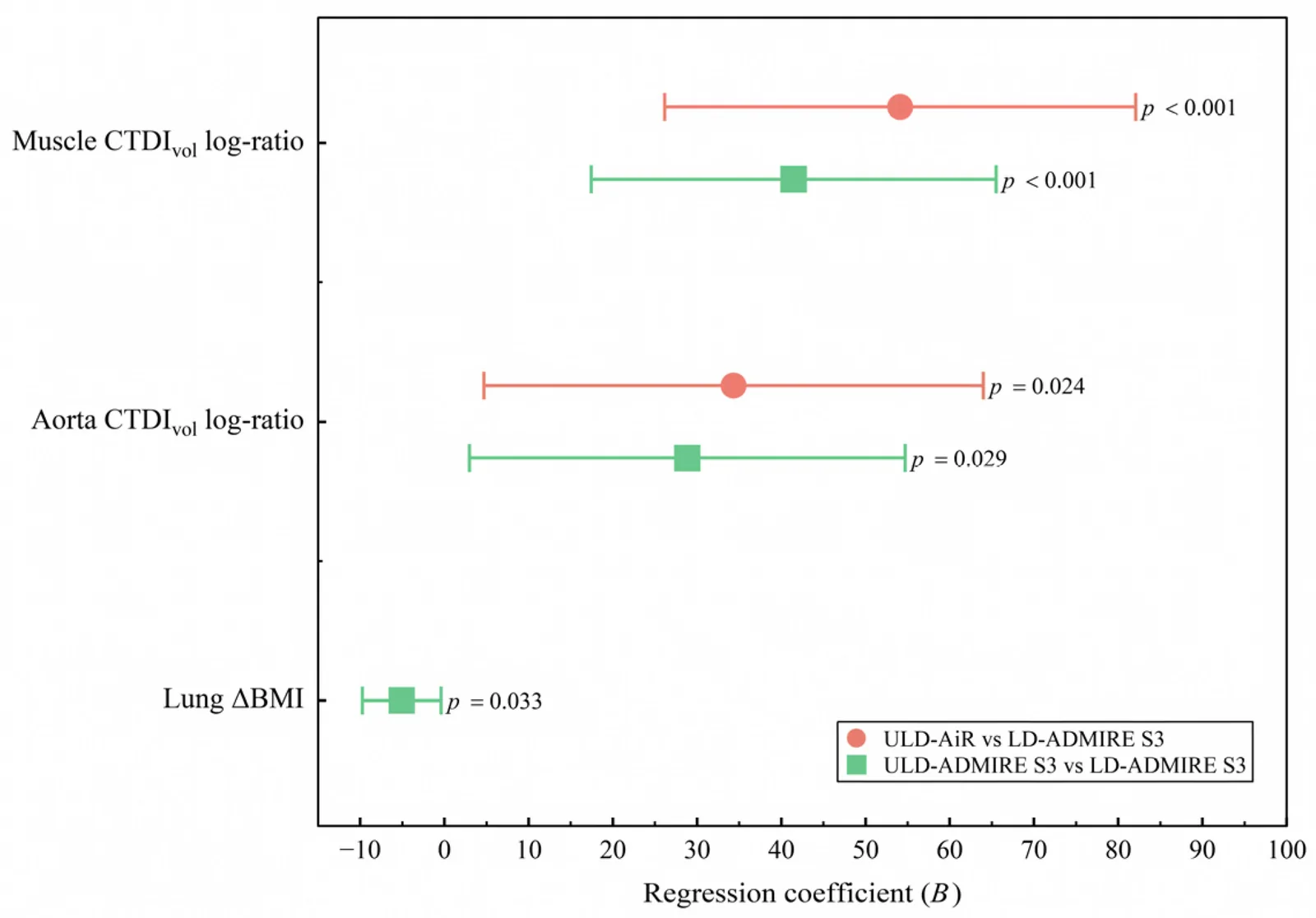
Selected associations from exploratory multivariable regression analyses of ULD-to-LD SNR log-ratios. Points represent regression coefficients (*B*), and horizontal bars represent 95% confidence intervals from the primary complete-case analysis (*n* = 169) for the two outcomes: ULD-ADMIRE S3 relative to LD-ADMIRE S3 and ULD-AiR relative to LD-ADMIRE S3. Only associations supported by the sensitivity analyses and, where applicable, the targeted influence analyses are displayed; complete regression results are provided in Supplementary Table S8. The aortic CTDI_vol_ associations should be interpreted cautiously because the corresponding overall models were not statistically significant. Regression *p*-values were two-sided, unadjusted, and exploratory. ADMIRE—Advanced Modeled Iterative Reconstruction; AiR—AiR Denoising; BMI—body mass index; CTDI_vol_—volume CT dose index; LD—low dose; SNR—signal-to-noise ratio; ULD—ultra-low dose.

The aortic CTDI_vol_ associations remained individually significant, although the corresponding overall models were not statistically significant (*p* = 0.077 for ULD-ADMIRE S3 and *p* = 0.198 for ULD-AiR), warranting cautious interpretation. The five associations shown in Figure 7 retained their coefficient directions and remained nominally significant in the 172-participant and multiple-imputation sensitivity analyses. Some secondary covariate estimates, particularly examination-interval terms, were sensitive to missing-data handling; associations that became significant only after imputation were not emphasized. Complete regression, sensitivity-analysis, and model-diagnostic results are provided in Supplementary Table S8, whereas the exploratory comparison of participants included and not included in the primary complete-case regression analysis is provided in Supplementary Table S9. Selected associations supported by the sensitivity analyses and, where applicable, the targeted influence analyses are shown in Figure 7.

## 4. Discussion

In this retrospective paired image-quality study, image-domain GAN-based denoising improved several objective and subjective image-quality metrics within both LD and ULD chest CT examinations. With a median paired reduction of 46.5% in estimated effective dose, ULD-AiR showed tissue- and endpoint-specific differences relative to LD-ADMIRE S3. In the lung, aorta, and muscle, ULD-AiR generally showed lower image noise and higher SNR/CNR, whereas liver findings were less consistent and vertebral quantitative metrics were less favorable. Subjectively, lung parenchyma scores showed no significant cross-dose difference, mediastinal soft-tissue scores favored ULD-AiR, and overall image-noise scores favored LD-ADMIRE S3. Exploratory multivariable analyses identified the CTDI_vol_ log-ratio as the most consistent correlate of the ULD-to-LD SNR log-ratio in the aorta and muscle, although the aortic model-level findings warrant cautious interpretation. Anthropometric associations were less consistent, and the muscle ΔBMI associations were sensitive to an influential observation. These findings are broadly consistent with previous low-dose CT studies reporting noise reduction and increased SNR/CNR with deep-learning-based image processing [9,10,11,19].

Although ULD-AiR showed lower objective image noise than LD-ADMIRE S3 in several tissues, subjective image-noise ratings favored LD-ADMIRE S3. This discordance suggests that ROI-based noise magnitude alone may not fully capture perceptual image quality. Differences in noise texture or other image characteristics not quantified in the present study may have contributed to the readers’ preferences; however, because noise power spectrum (NPS) and other task-based image-quality metrics were not evaluated, this explanation remains hypothetical. Previous studies have used NPS to characterize noise properties associated with deep-learning-based reconstruction [24,25]. Future studies combining NPS, spatial-resolution or task-based metrics, and reader assessment may help clarify the relationship between physical image characteristics and perceived image quality.

Recent studies have evaluated deep-learning-based reconstruction or image-domain post-processing at reduced chest CT dose levels. In our previous clinical study using the same AiR image-domain denoising approach on a different Siemens CT platform, AiR processing improved objective and subjective image-quality metrics of low-dose chest CT compared with Sinogram Affirmed Iterative Reconstruction (SAFIRE); however, comparison with standard-dose SAFIRE was descriptive because the two dose groups consisted of different patients and were acquired using different protocols [26]. Ye et al. reported improved image quality and pulmonary nodule detection with image-domain deep-learning processing of ULD CT using LD CT as the reference [14]. The present study extends this body of evidence by evaluating paired LD and ULD examinations on another CT platform and by directly characterizing tissue-specific objective and subjective image-quality differences between ULD-AiR and LD-ADMIRE S3. Previous studies have shown that the effects of deep-learning-based image processing may vary with algorithm type and reconstruction strength, radiation dose, reconstruction and display settings, and study population [9,10,16,27,28,29,30]. Such methodological heterogeneity may contribute to differences in the magnitude and tissue specificity of reported image-quality effects. The present findings therefore provide image-quality evidence for the evaluated Sn100 kVp protocol and fixed AiR configuration, but should not be interpreted as establishing diagnostic equivalence or generalizable screening performance.

Inter-reader reliability varied across tissues and quantitative metrics, with lower ICCs observed for several image-noise measurements, particularly in the liver and muscle. Because ROI-based SD measurements can be sensitive to local ROI placement and regional tissue heterogeneity, small reader-specific differences in ROI delineation may have contributed to this variability, although this mechanism was not directly evaluated in the present study [31]. In addition, ICC depends partly on between-subject variability; therefore, restricted measurement ranges can yield lower ICC values even when absolute measurement differences are limited [32]. Despite this variability, the direction of the principal AiR-related and cross-dose effects was generally concordant between the two readers. Subjective ratings showed consistently high inter-reader agreement using linear-weighted Cohen’s κ, supporting the reproducibility of the observed reader-level scoring patterns.

Our study has several limitations. First, this was a retrospective, single-center study performed on a single CT platform using one Sn100 kVp protocol, ADMIRE S3, and a fixed AiR setting, which limits generalizability to other scanners, protocols, and populations. Second, the paired LD and ULD examinations were acquired at separate time points (up to 3.39 years apart) and differed in acquisition parameters; therefore, residual temporal confounding cannot be excluded, and the cross-dose comparison should be interpreted as a protocol-level rather than an isolated dose comparison. Third, the study focused on ROI-based objective and subjective image-quality metrics without lesion detectability, diagnostic performance, NPS, or task-based assessments; thus, the findings do not establish diagnostic or screening equivalence. Fourth, objective ROI measurements were unblinded, and complete blinding during subjective assessment could not be guaranteed. Some implementation-level details of the proprietary AiR network also remain unavailable. Finally, exploratory regression analyses were based primarily on complete cases, and some associations were sensitive to missing-data handling or influential observations despite sensitivity analyses. Estimated effective dose was derived using a fixed chest-specific DLP conversion coefficient and should therefore be considered an approximate population-level estimate.

## 5. Conclusions

Image-domain GAN-based denoising improved several objective and subjective image-quality metrics within both LD and ULD chest CT examinations. With a median paired reduction of 46.5% in estimated effective dose, ULD-AiR showed tissue- and endpoint-specific image-quality differences relative to LD-ADMIRE S3. These findings provide image-quality evidence for the evaluated Sn100 kVp protocol and fixed AiR configuration but do not establish diagnostic or screening equivalence. Further prospective studies incorporating lesion-level and task-based performance assessment are warranted.

## Figures and Tables

**Table 1:** Radiation dose metrics for paired ULD and LD chest CT examinations (*n* = 262 participants).

Metric	LD CT, Median [IQR]	ULD CT, Median [IQR]	Paired Difference, Median (95% CI)	Median Paired Reduction [IQR]	*p* Value
CTDI_vol_ (mGy)	1.28 [1.15, 1.43]	0.68 [0.59, 0.79]	−0.60 (−0.62 to −0.57)	48.3% [40.8%, 53.0%]	<0.001
DLP (mGy·cm)	44.90 [40.70, 50.10]	25.25 [21.70, 29.30]	−20.45 (−21.60 to −19.40)	46.5% [38.7%, 51.5%]	<0.001
ED (mSv)	0.629 [0.570, 0.701]	0.354 [0.304, 0.410]	−0.286 (−0.302 to −0.272)	46.5% [38.7%, 51.5%]	<0.001

Note. Data are presented as median [interquartile range] unless otherwise indicated. Paired differences were calculated as ULD minus LD. The 95% confidence intervals for median paired differences were obtained using exact binomial order-statistic confidence intervals. Paired percentage reduction was calculated for each examination pair as 100 × (LD − ULD)/LD and is presented as median [interquartile range]. *p*-values were obtained using two-sided Wilcoxon signed-rank tests. CTDI_vol_—volume CT dose index; DLP—dose-length product; ED—estimated effective dose; CI—confidence interval; IQR—interquartile range; LD—low dose; ULD—ultra-low dose. ED was estimated as DLP × 0.014 mSv·mGy^−1^·cm^−1^.

## Data Availability

The data presented in this study are available on request from the corresponding author. The data are not publicly available due to privacy or ethical concerns.

## Supplementary Materials

The following supporting information can be downloaded at: https://www.mdpi.com/article/10.3390/diagnostics16182988/s1, Table S1. Subjective image-quality scoring criteria; Table S2. Paired CT-attenuation effect estimates for the three prespecified comparisons; Table S3. CT attenuation across the four-image series: paired comparisons and inter-reader reliability; Table S4. Image noise across the four-image series: paired comparisons and inter-reader reliability; Table S5. Signal-to-noise ratio across the four-image series: paired comparisons; Table S6. Contrast-to-noise ratio across the four-image series: paired comparisons; Table S7. Subjective image-quality scores across the four-image series: paired comparisons and inter-reader reliability; Table S8. Exploratory multivariable regression analyses of ULD-to-LD SNR log-ratios: primary complete-case analysis and sensitivity analyses; Table S9. Exploratory comparison of participants included and not included in the primary complete-case regression analysis.

## Abbreviations

The following abbreviations are used in this manuscript:

ADMIRE	Advanced Modeled Iterative Reconstruction
AiR	AiR Denoising
BMI	Body mass index
CI	Confidence interval
CNR	Contrast-to-noise ratio
CT	Computed tomography
CTDI_vol_	Volume CT dose index
DICOM	Digital Imaging and Communications in Medicine
DLIR	Deep-learning image reconstruction
DLP	Dose-length product
ED	Estimated effective dose
FBP	Filtered back projection
FDR	False discovery rate
GAN	Generative adversarial network
HU	Hounsfield unit
ICC	Intraclass correlation coefficient
IQR	Interquartile range
IR	Iterative reconstruction
LD	Low dose
NPS	Noise power spectrum
RESET	Regression equation specification error test
ROI	Region of interest
SAFIRE	Sinogram Affirmed Iterative Reconstruction
SD	Standard deviation
SNR	Signal-to-noise ratio
ULD	Ultra-low dose
